# Diagnostic challenge of mitral regurgitation caused by concomitant coronary obstruction and dynamic left ventricular outflow tract obstruction after transcatheter aortic valve replacement: a case report

**DOI:** 10.1093/ehjcr/ytag408

**Published:** 2026-06-04

**Authors:** Yuto Kawahira, Yuta Kato, Midori Miyazaki, Go Kuwahara, Shinichiro Miura

**Affiliations:** Department of Cardiology, Fukuoka University Hospital, 7-45-1 Nanakuma, Jyonan-ku, Fukuoka 814-0180, Japan; Department of Cardiology, Fukuoka University Hospital, 7-45-1 Nanakuma, Jyonan-ku, Fukuoka 814-0180, Japan; Department of Cardiology, Fukuoka University Hospital, 7-45-1 Nanakuma, Jyonan-ku, Fukuoka 814-0180, Japan; Department of Clinical Laboratory and Transfusion, 7-45-1 Nanakuma, Jyonan-ku, Fukuoka 814-0180, Japan; Department of Cardiovascular Surgery, 7-45-1 Nanakuma, Jyonan-ku, Fukuoka 814-0180, Japan; Department of Cardiology, Fukuoka University Hospital, 7-45-1 Nanakuma, Jyonan-ku, Fukuoka 814-0180, Japan

**Keywords:** Transcatheter aortic valve replacement, Coronary artery obstruction, Left ventricular outflow tract obstruction, Mitral regurgitation, Case report

## Abstract

**Background:**

Transcatheter aortic valve replacement (TAVR) is an established therapy for severe aortic stenosis (AS), but rare complications can rapidly become life-threatening, particularly in anatomically high-risk patients.

**Case summary:**

A 94-year-old woman with very severe AS underwent transfemoral TAVR using a self-expanding valve. After transcatheter heart valve implantation, she developed haemodynamic collapse with right coronary artery obstruction and severe mitral regurgitation (MR). At that time, the aetiology of the severe MR could not be clearly identified. Although intra-aortic balloon pump (IABP) was initiated, cardiac arrest occurred, requiring extracorporeal membrane oxygenation (ECMO). Percutaneous coronary intervention was unsuccessful, necessitating conversion to coronary artery bypass grafting. ECMO was discontinued on postoperative Day 1, but recurrent haemodynamic collapse developed under IABP support, revealing left ventricular outflow tract obstruction (LVOTO) and severe MR with systolic anterior motion (SAM). Discontinuing IABP and initiating medical therapy rapidly stabilized haemodynamics.

**Discussion:**

This case illustrates the diagnostic and haemodynamic complexity of simultaneous coronary artery obstruction and dynamic LVOTO with SAM-associated MR after TAVR. Because management strategies for these conditions are inherently contradictory, identifying the dominant mechanism of shock can be challenging. Although severe MR was initially attributed to ischaemia secondary to coronary obstruction, retrospective echocardiographic review demonstrated early SAM. Subsequent changes in loading conditions and myocardial contractility unmasked LVOTO. This case emphasizes the importance of repeated echocardiographic assessment and stepwise haemodynamic reassessment when multiple mechanisms of instability coexist after TAVR.

Learning points

- The coexistence of coronary obstruction and left ventricular outflow tract obstruction following transcatheter aortic valve replacement presents a clinical challenge, as the haemodynamic management strategies for each may be conflicting.
- Identifying the determinants of haemodynamic changes based on echocardiographic findings is important.

## Introduction

Transcatheter aortic valve replacement (TAVR) is an established treatment for severe aortic stenosis (AS),^1–4^ however, rare but life-threatening complications may still occur, particularly in anatomically high-risk patients.

We report a rare case of concomitant coronary obstruction and dynamic left ventricular outflow tract obstruction (LVOTO) with systolic anterior motion (SAM)-associated mitral regurgitation (MR) after TAVR, leading to recurrent haemodynamic collapse and highlighting the importance of repeated echocardiographic assessment.

## Summary figure

**Figure:**
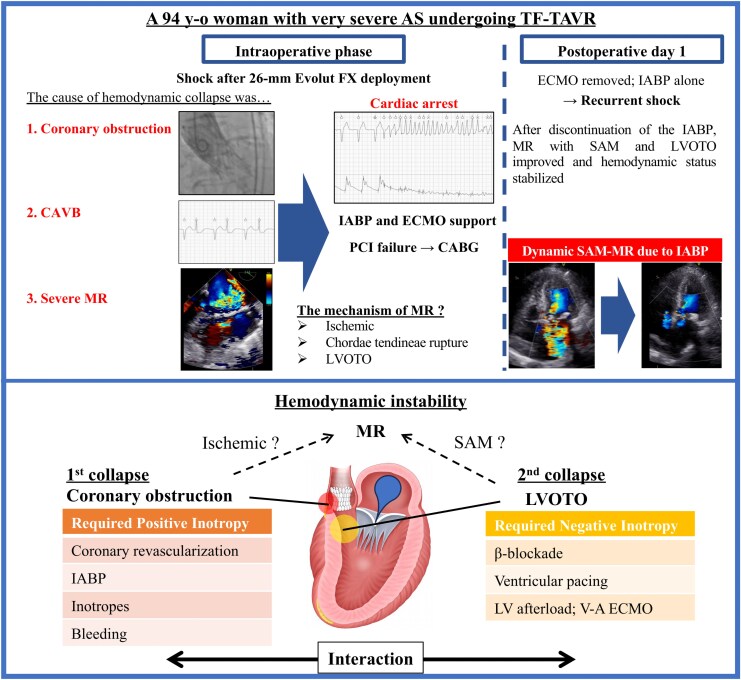


## Case presentation

A 94-year-old woman with New York Heart Association functional class III dyspnoea was referred for TAVR. Transthoracic echocardiography demonstrated preserved left ventricular ejection fraction with very severe AS (peak velocity 5.4 m/s, mean gradient 73 mmHg, aortic valve area 0.4 cm^2^), mild MR, and pulmonary hypertension (*Figure 1A,B*), without an intraventricular pressure gradient despite diffuse left ventricular hypertrophy. The patient had multiple comorbidities, including breast cancer, moderate chronic lung disease, and cerebrovascular disease, and was mildly frail (Clinical Frailty Scale 4). The Society of Thoracic Surgeons predicted risk of mortality was 26%. Preprocedural computed tomography (CT) revealed a type I bicuspid aortic valve with fusion of the right and non-coronary cusps with a calcified raphe (*Figure 1C*). The annulus area measured 352 mm^2^ with a perimeter of 68 mm. The right coronary artery (RCA) height was 10.9 mm. In addition, the right coronary cusp was characterized by a low sinus height (13.9 mm) and a small sinus of Valsalva diameter (25.0 mm). (*Figure 1D*). No significant coronary artery stenosis was observed, and vascular access was considered adequate for the *trans*-femoral approach. Considering the severity of AS, the presence of a heavily calcified bicuspid aortic valve, and a small annulus, we decided to perform TAVR using a self-expanding valve. The heart team noted the risk of coronary obstruction due to the height and diameter of the right sinus of Valsalva. Because the height of the RCA was deemed sufficient, coronary protection was not planned initially. Downsizing of the transcatheter heart valve (THV) was also considered; however, the risk of significant paravalvular regurgitation was judged to outweigh the risk of coronary obstruction. Trans-femoral TAVR was performed using a 26-mm Evolut FX valve (Medtronic, Dublin, Ireland) following pre-balloon aortic valvuloplasty (18-mm Inoue balloon, Toray Industries, Tokyo, Japan). No signs of coronary obstruction were observed during pre-balloon aortic valvuloplasty or deployment of the Evolut FX valve up to the point of no recapture (*Figure 2A*). Consequently, coronary protection was not performed, and the THV was fully released. Following full deployment of the Evolut FX, the patient developed complete atrioventricular block (CAVB), resulting in haemodynamic collapse. Transoesophageal echocardiography (TEE) revealed preserved left ventricular systolic function, and severe MR (*Figure 2B*). Aortography demonstrated RCA obstruction (*Figure 2C*). Although an intra-aortic balloon pump (IABP) was inserted, the patient remained haemodynamically unstable and subsequently experienced a ventricular fibrillation storm (*Figure 2D*), necessitating initiation of extracorporeal membrane oxygenation (ECMO). Percutaneous coronary intervention for RCA revascularization was attempted but unsuccessful due to difficulty in coronary cannulation; therefore, the strategy was converted to surgical coronary artery bypass grafting. Postoperatively, inotropes, including dobutamine (3–5 μg/kg/min) and noradrenaline (0.1–0.2 μg/kg/min), along with mechanical circulatory support with ECMO (flow 3.0 L/min) and IABP, reduced MR and resulted in haemodynamic stabilization. The patient was weaned from ECMO on postoperative Day 1; however, haemodynamic shock occurred within a few hours after decannulation. At that time, transthoracic echocardiography revealed recurrent MR caused by LVOTO with SAM (*Figure 3A, B*). After discontinuation of the IABP, MR with SAM and LVOTO improved and hemodynamic status stabilized (*Figure 3C, D*). The cause of haemodynamic shock was determined to be acute severe MR associated with SAM and LVOTO, which became apparent following IABP. Accordingly, IABP support and inotropes were discontinued, medical therapy with a β-blocker and cibenzoline was administered. Postoperative CT demonstrated patent bypass grafts; however, the RCA ostium was completely occluded due to calcification of the right coronary cusp leaflet (*Figure 4*). On postoperative Day 53, the patient was discharged and transferred to another hospital for further care in a haemodynamically stable condition without significant neurological deficits.

**Figure 1:**
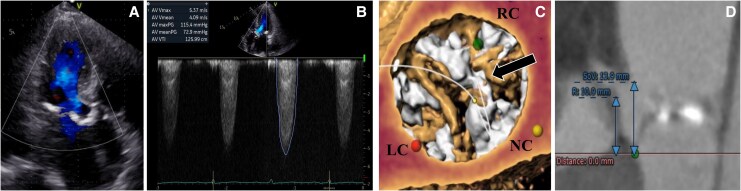
Preprocedural transthoracic echocardiography and computed tomography preprocedural transthoracic echocardiography showed very severe aortic stenosis with mild mitral regurgitation (*A*, *B*). Computed tomography demonstrated a type I bicuspid aortic valve with fusion of the right and non-coronary cusps and a calcified raphe (arrow, *C*), as well as low right sinus height (*D*).

**Figure 2:**
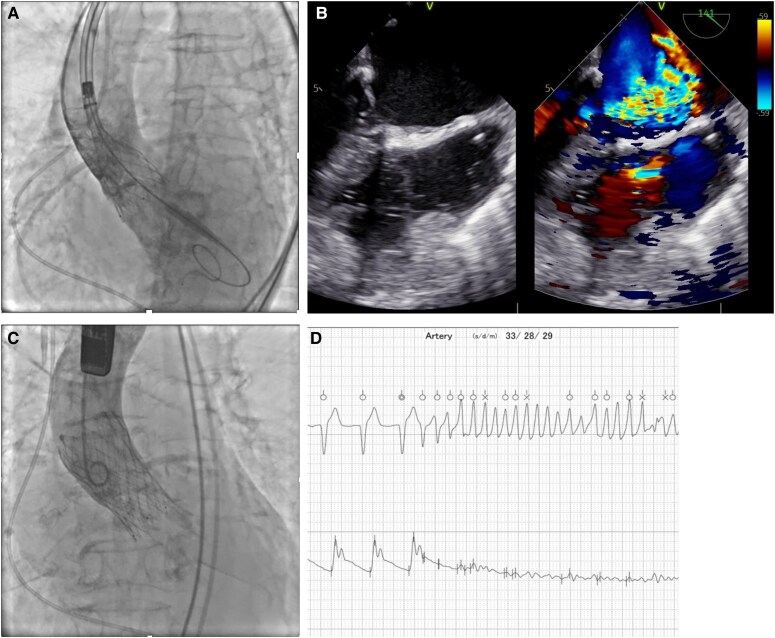
Intraoperative aortography and transoesophageal echocardiography. Aortography in the left anterior oblique view showed Evolut FX deployment to the point of no recapture without coronary obstruction (*A*). During subsequent haemodynamic collapse, transoesophageal echocardiography revealed severe mitral regurgitation (*B*), and aortography demonstrated right coronary artery obstruction (*C*). Percutaneous coronary intervention failed, followed by a ventricular fibrillation storm (*D*).

**Figure 3:**
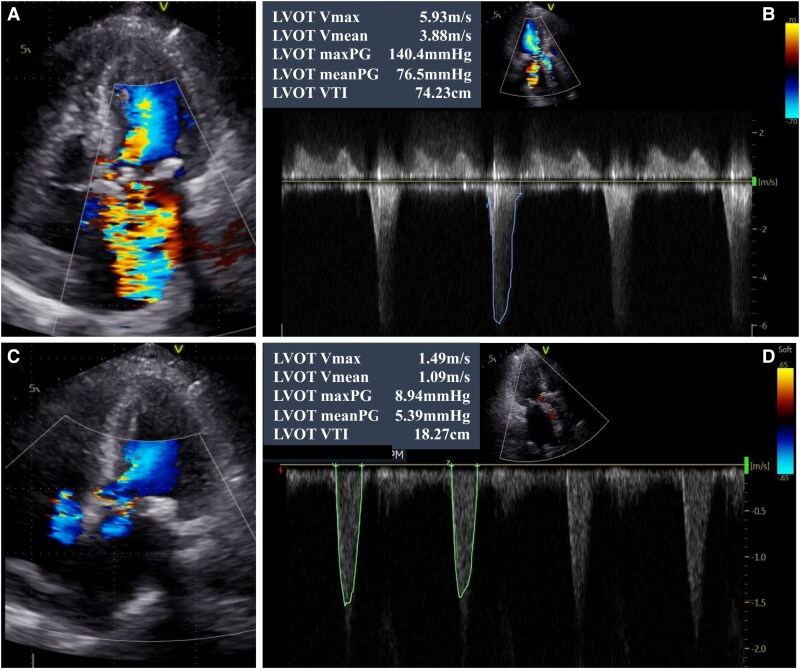
Transthoracic echocardiography on postoperative Day 1. After extracorporeal membrane oxygenation removal under intra-aortic balloon pump support, left ventricular outflow tract obstruction with systolic anterior motion and mitral regurgitation became evident (*A*, *B*), but improved after balloon pump discontinuation (*C*, *D*).

**Figure 4:**
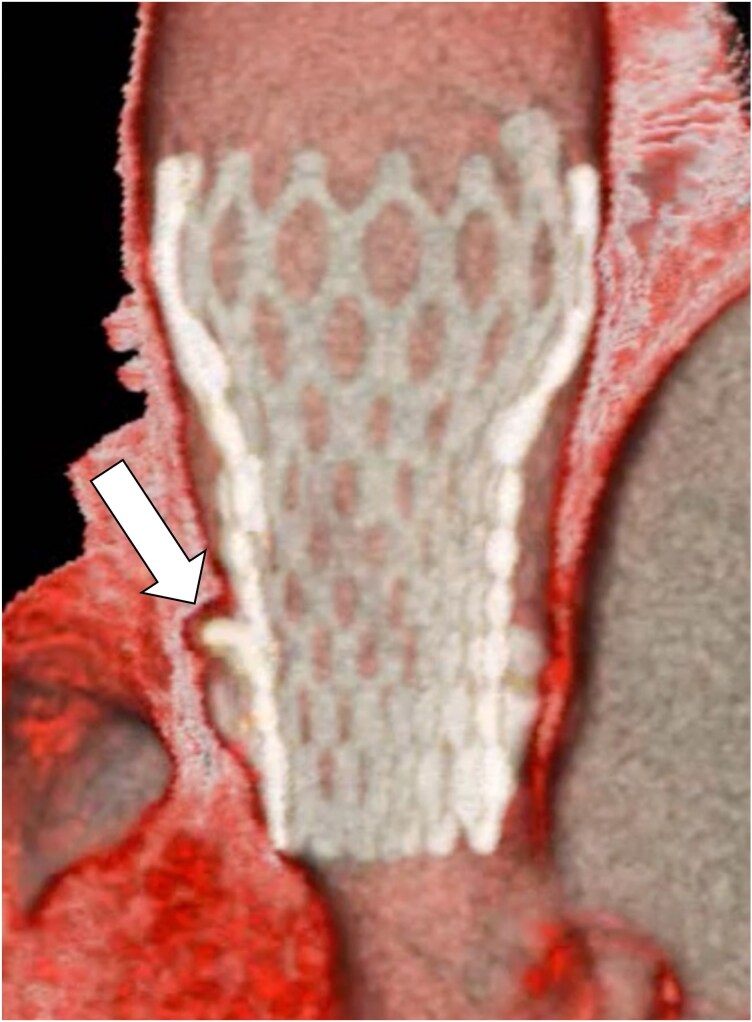
Postprocedural computed tomography. Postprocedural computed tomography showed complete occlusion of the right coronary artery ostium due to calcification of the right coronary cusp leaflet (arrow).

## Discussion

This case involved two distinct episodes of haemodynamic collapse following TAVR, each with different primary mechanisms. The primary cause of the initial collapse immediately after THV deployment was acute RCA obstruction. This led to CAVB, resulting in rapid haemodynamic deterioration. At that time, coronary obstruction and conduction disturbance were considered the main drivers of circulatory collapse. The second episode of haemodynamic collapse occurred after weaning from ECMO, under support with IABP and inotropes, and was primarily attributed to dynamic LVOTO with SAM leading to severe MR. This mechanism became apparent under altered loading conditions, including reduced preload and changes in afterload and contractility. Retrospective review of intra-procedural TEE revealed that LVOTO with SAM was already present at the onset of the initial collapse. However, due to the need for immediate cardiopulmonary resuscitation and haemodynamic stabilization, detailed assessment of the mechanism of MR was not feasible. These findings highlight the importance of recognizing that multiple, sequential mechanisms may contribute to haemodynamic collapse after TAVR. While coronary obstruction may act as the primary trigger, dynamic LVOTO with SAM can coexist or become unmasked during the clinical course, particularly under changing haemodynamic conditions.

## Conclusion

Coronary obstruction following TAVR may initiate a complex haemodynamic cascade, in which dynamic LVOTO with SAM leading to MR can coexist or become unmasked during the clinical course. Careful evaluation should include both ischaemic and dynamic mechanisms when assessing unexplained haemodynamic collapse after TAVR.

## Data Availability

The data underlying this article cannot be shared publicly due to patient privacy but are available from the corresponding author upon reasonable request.

## Lead author biography

Dr Yuto Kawahira was born on 12 August 1990. He graduated from Fukuoka University in 2016 and completed his initial residency training at Fukuoka University Hospital from 2016 to 2018. In 2018, he joined the Department of Cardiovascular Medicine at Fukuoka University Hospital. He is currently engaged in both clinical practice and research, with a primary focus on structural heart disease and transcatheter cardiovascular interventions.
